# Comparison of two sources of iodine delivery on breast milk iodine and maternal and infant urinary iodine concentrations in southern Ethiopia: A randomized trial

**DOI:** 10.1002/fsn3.477

**Published:** 2017-04-07

**Authors:** Tafere Gebreegziabher, Barbara J. Stoecker

**Affiliations:** ^1^ Hawassa University Hawassa Ethiopia and Central Washington University Ellensburg Washington USA; ^2^ Oklahoma State University Stillwater Oklahoma USA

**Keywords:** Breast milk iodine concentration, Ethiopia, maternal iodine intake

## Abstract

Iodine deficiency during pregnancy and lactation could expose the infant to severe iodine deficiency disorders. A randomized supplementation trial among rural lactating women was conducted in Sidama zone, southern Ethiopia, to compare the methods of iodine delivery on breast milk iodine, and on maternal and infant urinary iodine concentrations. Women were randomly assigned either to receive 225 μg iodine as potassium iodide capsule daily for 6 months or 450 g of appropriately iodized salt (30–40 μg I as KIO_3_/g of salt) weekly for household consumption for 6 months. Breast milk iodine concentration (BMIC) and maternal and infant urinary iodine concentration (UIC) were measured at baseline and at 6 months. The women did not differ in BMIC and UIC, and infants did not differ in UIC in a time by treatment interaction. Median (IQR, interquartile range, IQR) BMIC at baseline was 154 [43, 252] μg/L and at 6 months was 105 [36, 198] μg/L, maternal UIC at baseline was 107 [71, 161] μg/L and at 6 months was 130 [80, 208] μg/L; infant UIC at baseline was 218 [108, 356] μg/L and at 6 months was 222 [117, 369] μg/L. Significant correlations among the three variables were obtained in both groups at both times. We conclude that for lactating women an adequate amount of appropriately iodized salt (30–40 μg I/g) had similar effects as a daily supplement of 225 μg I on BMIC and on maternal and infant UIC.

## 1 INTRODUCTION

Iodine is required for the synthesis of thyroid hormones; thyroid hormones in turn regulate the metabolic patterns of cells. Iodine plays a crucial part in the process of early growth and development of most organs, especially the brain (Chan & Kilby, 2000). Severe maternal iodine deficiency during early pregnancy could result in permanent brain damage to the offspring (Halpern et al., 1991; Pharoah, Buttfield, & Hetzel, 1971). Mild‐to‐moderate iodine deficiency may result in reduced learning ability, and increased risk of low birth weight and of infant morbidity and mortality (Zimmermann, 2009). Infants under two years of age are among the most vulnerable groups to be affected by iodine deficiency along with pregnant and lactating mothers (WHO/UNICEF 2007). Hence, special attention is needed for these population groups regarding preventing and controlling iodine deficiency (Delange, 2004).

Risk of iodine deficiency in early infancy is high even in iodine sufficient areas because infant intrathyroidal reserve turnover rate is rapid (Delange, 1998). Therefore, in iodine insufficient areas, recommendations for iodine for women during pregnancy and lactation are increased to 250 μg/day or to a 400 mg single annual dose of iodized oil supplement (Andersson, de Benoist, Delange, & Zupan, 2007). Because a large amount of iodine is secreted into breast milk, exclusively breastfed infants 0–6 months of age should receive sufficient iodine through breast milk (Delange, 2004; Spitzweg, Joba, Eisenmenger, & Heufelder, 1998).

Recommended intakes for infants are based on mean iodine intake of healthy full‐term infants fed breast milk, because functional criteria that reflect iodine intake in infants are not available. Hence, the WHO recommends daily intake of iodine for infants as 90 μg (WHO/UNICEF/ICCIDD 2007). Similarly, the Institute of Medicine (IOM) in the USA set the adequate iodine intake for infants 0–6 months of age at 110 μg/day. This recommendation is based on two important points. First, the median breast milk iodine concentration (BMIC) in the United States was 146 μg/L (Institute of Medicine 2001). Second, based on the estimated daily breast milk excretion, the mean amount of iodine obtained by an infant from 0.78 L of human milk is approximately 115 μg/day (Institute of Medicine 2001). Iodine intake greater than the requirement generally will be excreted in the urine (Zimmermann, 2007).

BMIC can be increased by increasing maternal iodine intake (Zimmermann, 2007). In a study in 16 healthy lactating US women, the BMIC was significantly increased by a one‐time ingestion of 600 μg potassium iodide (456 μg of iodine) with peak levels at 6 hr (Leung, Braverman, He, Heeren, & Pearce, 2012). In a randomized, double‐blind, placebo‐controlled study, supplementation to lactating women of either 75 μg/day or 150 μg/day of iodine as potassium iodate for 6 months significantly increased the BMIC and maternal urinary iodine concentration (UIC). However, the amount of supplementation given was not sufficient to improve the mothers’ or infants’ thyroid hormones (Mulrine, Skeaff, Ferguson, Gray, & Valeix, 2010). In another double‐blind, randomized, placebo‐controlled trial, one dose of 400 mg of iodine as a capsule of iodized poppy seed oil to lactating mothers significantly increased the UIC of their 3‐month‐old infants compared with a single dose of 100 mg iodine given orally as an iodized oil supplement directly to the infants (Bouhouch et al., 2014).

As a strategy to alleviate iodine deficiency, increasing iodine intake through supplementation or food fortification has been recommended. Salt iodization programs have been found to be most useful because they are cost‐effective and feasible, and salt is consumed by almost everyone (WHO/UNICEF/ICCIDD 2007). In places where salt iodization is not feasible and/or is unavailable, iodine supplements should be given to at‐risk groups including pregnant or lactating women and infants (Andersson et al., 2007).

Previous studies in rural Sidama reported median UIC of 15 μg/L in pregnant women (Ersino, Tadele, Bogale, Abuye, & Stoecker, 2013) and 37 μg/L in reproductive age women (Gebreegziabher et al., 2013). Our study was designed and approved before Ethiopia's salt iodization legislation was enacted. Once the legislation was passed, program implementation was rapid and the population began to have access to iodized salt. Thus, our study with lactating women compared the efficacy of appropriately iodized household salt to daily intake of iodine capsules in raising the BMIC and maternal and infant UIC in a context of villagers already having some access to iodized salt.

## 2 MATERIALS AND METHODS

### 2.1 Data source and study population

The study was conducted in rural areas of Sidama zone, southern Ethiopia. All lactating women (*n* = 101) in the randomly selected study villages who delivered between January and February, 2013 were recruited within a week after delivery to participate in the study. Informed consent was obtained from the women for themselves and for their infants. This study was conducted in accordance with the ethical principles for the protection of human subjects. Ethical approval was obtained from Oklahoma State University (OSU), USA, Hawassa University, Ethiopia, and the Ministry of Science and Technology, Ethiopia. The study was a randomized intervention trial.

Mother‐infant dyads were assigned using random numbers to receive either 225 μg of iodine as potassium iodide in capsule form (Pure Encapsulations, Inc. Boston, MA) administered to the mother daily for 6 months or 450 g (30–40 μg of I as KIO_3_/g of salt) of iodized salt (I‐salt) delivered weekly for household consumption for 6 months.

Mean iodine concentration of the salt manufactured for this research project was verified by inductively coupled plasma mass spectrophotometry (ICP‐MS, Elan 9000, Perkin‐Elmer, Norwalk, CT) to be 35.1 (3.6) μg/g, consistent with the recommendation (20–40 mg of iodine per kg of salt) of international organizations (WHO/UNICEF/ICCIDD 1996, 2007). The dose of 225 μg of iodine capsule administered daily was at the lower end of the recommended range for lactating women (225–350 μg daily) (Delange, 2007).

Capsules were administered to the mothers daily by health extension workers or salt for the household was delivered in a sealed plastic bag weekly. The amount of salt remaining at the end of the first week was weighed to estimate per capita consumption and the mean was 10.3 (2) g. Women were strictly reminded every week not to buy or use salt from the local market until the end of the study and not to share the salt we gave them with anybody outside their household. However, the culture of valuing and sharing salt may have reduced the amount of leftover salt reported.

### 2.2 Anthropometry and questionnaire

Weight (kg) and height (cm) of mothers were measured to calculate body mass index (BMI = weight (kg)/height (m)^2^). Weight was measured on a solar digital scale (Uniscale, UNICEF, NY) and recorded to the nearest 100 g and height was measured to the nearest 0.1 cm using a single calibrated instrument (Adult Board, Schorr Productions, Olney, MD). Mid upper arm circumference (MUAC) was measured to the nearest 0.1 cm using a non‐stretchable plastic measuring tape. A pre‐tested questionnaire was administered to assess the socioeconomic and demographic characteristics including household size of the mothers participating in the study.

Before delivery of supplements, baseline data including anthropometry, urine samples, breast milk samples, and sociodemographic information were collected from mothers. Infant urine samples also were obtained. Following 6 months of supplementation, the same biological samples were collected from mothers and their infants.

### 2.3 Biochemical measurements

#### 2.3.1 Breast milk iodine

Ten mL of breast milk was collected from each mother in plastic cups and stored in tightly capped vials. Breast milk samples were collected between 9:00 and 11:00 AM. Samples were frozen until they are analyzed. To extract iodine from the breast milk 1.5 mL of 25% tetramethylammonium hydroxide (TMAH) solution (Sigma‐Aldrich, St. Louis, MO) and 0.5 mL of 30% hydrogen peroxide were added to the breast milk samples (3 mL) and mixed well. These samples were incubated in an oven at 90°C for 3 hr using DigiTUBES (Perkin‐Elmer, Waltham, MA). After cooling to room temperature, the samples were centrifuged at 1,532 x g for 15 minutes. For analysis, all breast milk samples were diluted 25‐fold with 5% TMAH (Sigma‐Aldrich, St. Lowis, MO) in deionized water. BMIC was analyzed by ICP‐MS (Fecher, Goldmann, & Nagengast, 1998). Tellurium (Perkin‐Elmer Life and Analytical Sciences, Shelton, CT) was utilized as an internal standard. Non‐fat milk powder (RM 1549, National Institute of Standards and Technology, Gaithersburg, MD) was used as an external control. The reference value for the external control was 3.38 ± 0.02 mg I/kg and the measured value was 3.35 ± 0.03 mg/kg.

### 2.4 Urinary iodine

Mothers provided fresh urine in a cup from which samples were transferred to vials that were then tightly sealed. Urine samples were collected from infants by placing cotton balls appropriately inside a plastic sheet available in the local market. The infants were checked every 10 minutes until urine was obtained. Urine samples were collected by squeezing urine out of the cotton balls. New trace mineral free gloves were used for each infant to avoid contamination. Moreover, samples of cotton balls and plastic sheets were soaked in deionized water and the iodine content in the water was assayed. Iodine content in the water was below the detection limit of 1 μg/L.

Samples were frozen at −20°C at Hawassa University and then transported to OSU for analysis. Urinary iodine was diluted in 2% ammonium hydroxide and analyzed by ICP‐MS, with tellurium as internal standard (Caldwell et al., 2003). Quality control samples for urinary iodine were measured every 10 samples to ensure stability of the instrument. Urinary creatinine was analyzed using a BioLis 24i clinical chemistry analyzer with standard reagents (Carolina Liquid Chemistries Corp., Brea, CA).

### 2.5 Salt and drinking water iodine concentration

A 10 g salt sample was collected at baseline in a tightly sealed plastic bag from each household for iodine content analysis. Salt iodine concentration (SIC) was analyzed using ICP‐MS. A salt sample (1 gm) was diluted with 2% NH_3_OH (Sigma‐Aldrich, St. Louis, MO) in deionized water and tellurium was utilized as an internal standard.

Drinking water samples were collected from each of the study villages. Water iodine concentration was analyzed using the same method as UIC.

### 2.6 Statistical analysis

The primary outcome variables in this study were the BMIC and maternal and infant UIC. All data were checked for normality using the Kolmogorov‐Smirnov test and skewed data were log transformed before analyses.

The PROC MIXED command was used for linear mixed model analysis to assess the effect of the two methods of iodine supplementation at 6 month. The model tested treatment (between groups) and time (within groups) and treatment * time interaction.

Associations between outcome variables were assessed using Spearman's correlation analysis. The relation between multiple variables and infant UIC were modeled using stepwise linear regression.

Data are reported as mean (SD), median with interquartile range [IQR], or frequency (%) as appropriate. Significance was set at *p* < .05. SPSS (version 20; IBM Corp. Armonk) and SAS (Version 9.3; SAS Institute Inc., Cary, NC) were used for data analyses.

## 3 RESULTS

### 3.1 Characteristics of study participants at baseline

All women breastfed exclusively in the first week after delivery and continued breast feeding to 6 months. However, only 58% reported exclusive breast feeding at the age of 6 months (data not shown). The mean (SD) times per day of breast feeding in the 24 hr preceding the survey at first week in both groups was 12.8 (5.2) (Table 1).

**Table 1:** Socio‐demographic and anthropometric characteristics of mother‐infant dyads at baseline (*n* = 101)

	Capsule group (*n* = 50)	I‐Salt group (*n* = 51)	*p*‐value
Mothers			
Age (years)	23 [20, 27]	21 [20, 25]	.14
MUAC (cm)	23.2 (1.8)	23.3 (1.7)	.62
BMI (kg/m^2^)	21.2 (2.5)	21.8 (2.0)	.15
Gravidity	2 [1.75, 4]	2 [1, 5]	.41
Parity	2 [1, 4]	2 [1, 4]	.38
School years	2 [2, 3]	2 [1, 3]	.42
Household size	6 (2.6)	5.7 (2.0)	.94
Frequency of breast feeding yesterday (night hours)	6.7 (2.5)	6.4 (3.1)	.35
Frequency of breast feeding yesterday (daylight hours)	6.5 (2.5)	6.0 (3.1)	.28
Infants			
Age (days)	5 [3, 7]	6 [5, 8]	.06
Sex			.765
Male	48% (24/50)	51% (26/51)	
Female	52% (26/50)	49% (25/51)	

Data are median [IQR], mean (SD), % (*n*/N). Group mean difference was analyzed using *T*‐test or Mann‐Whitney *U* test, and the categorical variable (sex) was analyzed using the Chi‐square test, *p* < .05 was considered significant.

Mother‐infant dyads did not differ in any sociodemographic characteristics assessed as indicated in Table 1. The median [IQR] age of all mothers was 22 [20, 25] years, mean BMI was 21.5 (2.3) kg/m^2^ and MUAC was 23.3 (1.7) cm. The mean gravidity and parity of all the mothers were 3 (2) and the mean household size was 6.0 (2.3). Mean school attendance was 3.8 (3.5) years.

The median age of infants at baseline was 6 [4, 8] days. There were 50 male and 51 female infants enrolled in the study. The average household salt use per week was 440 (2) g and no household reported using less than 435.5 g during the first week.

### 3.2 Breast milk and urinary iodine concentration of mothers and their infants

Table 2 shows baseline and 6 months concentrations and distributions of BMIC and of UIC of mothers and their infants. The women were not significantly different in BMIC or UIC by treatment and the infants were not significantly different in the UIC at the two time points for either treatment. To better understand effects of daily iodine supplementation, we categorized BMIC into different groups. At the end of the study at 6 months 36% of the women had BMIC < 60 μg/L, 18% had 60–120 μg/L, 29% had 120–240 μg/L and 16% had >240 μg/L. BMIC was lower at 6 months than at baseline but there was no significant time by treatment interaction (*p* = .656).

**Table 2:** BMIC and UIC of mothers and infants in subjects who received iodine as a capsule (*n* = 50) or iodized salt (*n* = 51)^a^

	Capsule group		I‐salt group	
Mothers	Baseline	6 months	Baseline	6 months
BMIC (μg/L)^b^	149 [46, 266]	104 [39, 197]	157 [29, 243]	111 [34, 202]
BMIC < 120 (μg/L)^c^	40%	56%	39%	53%
UIC (μg/L)	136 [76, 173]	150 [86, 220]	95 [64, 142]	110 [73, 191]
UIC < 100 (μg/L)	42%	32%	53%	45%
I/Cre (μg/g)	214 [142, 292]	176 [126, 383]	203 [139, 268]	173 [117, 292]
Infants				
UIC (μg/L)	234 [121, 379]	254 [130, 400]	193 [107, 331]	195 [108, 352]
UIC < 180 μg/L^d^	40%	38%	49%	47%
UIC < 100 μg/L	16%	8%	18%	24%

a Data are median [IQR] or percentage.

b BMIC, breast milk iodine concentration; UIC, urinary iodine concentration; Cre, creatinine; I‐salt group, iodized salt group.

c BMIC ≥ 120 μg/L is assumed to provide the infant's daily requirement of 90 μg (Zimmermann, 2007).

d Infant UIC ≥ 180 μg/L represents the expected UIC output in the neonates and infants when the recommended dietary intake of iodine (225–350 μg/day) for lactating mothers and 90 μg of iodine daily for neonates and infants are met (Delange, 2007).

According to the WHO classification for iodine deficiency in lactating women, 47.5% and 38.6% of the women had UIC < 100 μg/L at baseline and at 6 months, respectively. Furthermore, at baseline 11% of the women had UIC below 50 μg/L and 10% were below 50 μg/L at 6 months. Maternal UIC showed no time by treatment interaction (*p* = .668). Also, maternal UIC adjusted for creatinine was not significantly different between groups or over time (*p* = .748).

The proportion of infants with the UIC < 100 μg/L at baseline was 20.8% and at 6 months was 15.8%. The median UIC of infants was not significantly different in time by treatment interaction (*p* = .322) (Table 2).

Household salt iodine concentration collected at baseline showed high variation (0–42 μg/g) with a median [IQR] of 8.1 [4.3, 13.4] μg/g. Among the salt samples tested 21% met the minimum recommendation (15 μg/g) (WHO/UNICEF/ICCIDD 2001) for iodine adequacy (Data not shown). Drinking water iodine concentration was below the detection limit of 1 μg/L.

Relations between BMIC, maternal UIC and infant UIC at 6 months were examined (Data not shown). BMIC was significantly correlated with maternal UIC (r = .39, *p* < .001) and infant UIC (r = .44, *p* < .001). The correlation between BMIC and maternal UIC increased when maternal UIC was adjusted for creatinine (r = .51, *p* < .001). Maternal UIC was correlated with infant UIC (r = .31, *p* < .001). The correlation coefficients at baseline were similar to those at 6 months.

In Table 3, multiple regression models with four best‐fitting variables predicting infant UIC are presented. The model at baseline with all four predictors produced an adjusted *R* ^*2*^ = .290 and at 6 months the model adjusted *R* ^*2*^ was .316. At baseline maternal UIC/creatinine, BMIC and infant sex had significant regression weights, indicating infants whose mothers had higher maternal UIC and BMIC were expected to have higher UIC and female infants were expected to have lower UIC than males. At 6 months maternal UIC/creatinine and BMIC had significant regression weights, indicating that higher maternal UIC and higher BMIC were expected to be associated with higher infant UIC.

**Table 3:** Multiple regression predicting UIC of infants at baseline and 6 months^a^

Variables	At baseline			At 6 months		
	β	95% CI	*p*	β	95% CI	*p*
Maternal UIC/Creatinine (μg/g)	0.30	0.15, 0.64	.002	0.42	0.26, 0.65	.000
BMIC (μg/L)	0.26	0.05, 0.29	.006	0.27	0.06, 0.28	.003
Infant's age (days)	−0.16	−0.05, 0.003	.083	0.12	−0.01, 0.07	.167
Female infant^b^	−0.18	−0.24, −0.002	.046	−0.13	−0.19, 0.02	.121
Adjusted R‐square	0.290			0.316		

a The dependent variable (infant UIC) and the independent variables including maternal UIC/Creatinine and BMIC were analyzed as log transformed data because they were not normally distributed (*n* = 101).

b Infants sex was coded as 0 for Female and 1 for Male.

## 4 DISCUSSION

This study showed BMIC, maternal UIC and infant UIC were not significantly different between mothers who received 225 μg of iodine daily as a capsule for 6 months in addition to variable amounts of iodine (median 8 [4.3, 13.4] μg/g) in their existing household salt and mothers who received 450 g per household of appropriately iodized salt (30–40 μg/g) delivered weekly for 6 months.

Average (SD) salt intake in Ethiopia nationally was estimated at 8.4 (5.9) g while intakes in the study region were estimated at 17.2 (13.8) g per person per day (Abuye, Berhane, Akalu, Getahun, & Ersumo, 2007). One reason the salt intake in this region is high is because people in the rural areas consume salt in their coffee several times a day in addition to other food items. From our weekly follow up however, the amount of salt we provided (450 g) was reported to be sufficient for the household for 1 week. The mean amount of salt remaining at the end of the first week was 10.3 g.

In the current study, the average household size in the I‐salt group was 5.7. However, because one of the household members was a new born infant, who would not be using salted foods, the daily discretionary per capita salt intake was 13.7 g based on a household size of 4.7. This amount of salt with mean iodine content of 35.1 μg/g would provide a calculated mean discretionary intake of 481 μg of iodine per person per day. Even with cooking losses which have been estimated at 20%, the iodine intake would exceed the 250 μg daily recommended for lactating women (Delange, 2004, WHO/UNICEF 2007).

Maternal iodine intake is critical during lactation in order to provide the breastfeeding infant's iodine requirement. Iodine is concentrated through the sodium iodide symporter mechanism, which promotes increased expression of iodine in milk during lactation (Tazebay et al., 2000). Thus, iodine is present in breast milk at higher concentrations than in plasma (Azizi & Smyth, 2009).

The median BMIC (154 [43, 252] μg/L) at baseline was within the normal range of 150–180 μg/L in iodine sufficient countries (Dorea, 2002; Semba & Delange, 2001). Despite iodine supplementation of 225 μg daily or well‐iodized salt weekly for 6 months, median BMIC decreased from 154 μg/L at baseline to 105 [36, 198] μg/L at 6 months. Consistent with our study, although the baseline iodine status of the women in New Zealand was lower, Mulrine et al. (2010) found that BMIC declined over the first 24 weeks in non‐supplemented lactating women and supplementation with 75 μg or 150 μg of iodine daily did not increase BMIC to a level that could raise infant median UIC. In the women who received 75 μg of iodine for 24 weeks, BMIC ranged between 35 to 57 μg/L and median infant UIC was 50 [22, 60] μg/L. In the group who received 150 μg, BMIC ranged between 43 and 70 μg/L and infant UIC was 66 [36, 87] μg/L (Mulrine et al., 2010). In another study, BMIC in non‐supplemented mothers declined from 43 μg/L at baseline to 26 μg/L over a 9‐month‐period while milk from the mothers supplemented with 400 mg of iodine declined from 41 μg/L at baseline to 39 μg/L at 9 months (Bouhouch et al., 2014).

It should be noted that our baseline data were collected within 1 week (with more than 32% within 4 days) after delivery, when some colostrum was still available. Iodine concentration is high in colostrum and decreases over time, which could contribute to BMIC decrease from baseline to 6 months (Semba & Delange, 2001). On the other hand, volume of milk consumed increases substantially between the first days after birth and 6 months of age, so the total amount of iodine received by the infant at 6 months would not have decreased (Neville et al., 1988, WHO 1998). However, because the decrease in iodine concentration in breast milk over time has not been measured in detail, it is difficult to quantify the effect our supplementation had on the usual decrease in BMIC from colostrum to mature milk.

A study conducted at a time when only 4.2% of households in Ethiopia consumed iodized salt reported BMIC in the range of 5–16 μg/L (Abuye & Kelbessa, 2000). Our data collected after the policy that all salt should be iodized showed a wide range in BMIC at baseline as well as at 6 months. At baseline, the BMIC ranged from 8 μg/L to 965 μg/L with 40% below 120 μg/L. At 6 months the range was from 4 μg/L to 958 μg/L with 57% below 120 μg/L. Only 10% of the women had BMIC > 300 μg/L in response to supplementation. Although there was wide range in both BMIC and UIC, there were few concerningly high values in response to either iodized salt or iodine capsule and no adverse reaction was reported in any of the study participants. In a study of 50 Korean women, whose diets frequently included seaweed soup, considerable elevation and range of BMIC from 198 to 8484 μg/L was reported (Rhee, Braverman, Pino, He, & Pearce, 2011). Significant variation of BMIC ranging from 5 to 2170 μg/L (Dorea, 2002) and from 9 to 1267 μg/L (Semba & Delange, 2001) were also reported in two other reviews. This variation indicates that many infants may not get adequate amounts of iodine to meet their requirement while others consume large amounts of iodine without apparent adverse reaction.

Maternal median UIC was not significantly different between the two types of iodine supplements. Based on the range recommended by Delange (2007), median maternal UIC of 150–230 μg/L as an indicator for optimal iodine nutrition during pregnancy and lactation, most of our study participants in both groups fell below the minimum value at baseline. Following 6 months of iodine supplementation, however, most of the women in the capsule group were within the minimum range whereas most of the women in the I‐salt group remained below the range.

For women in our study who had UIC > 100 μg/L, which also has been considered adequate for lactating women, 16% at baseline and 29% at 6 months had BMIC < 120 μg/L, which is the concentration assumed adequate to cover the infant's daily requirement of 90 μg (Zimmermann, 2007). For all women (*n* = 101) combined at 6 months, those mothers with UIC below 150 μg/L (*n* = 54) had median BMIC of 83 μg/L and above 150 μg/L (*n* = 47) median BMIC was 133 μg/L. This could indicate that iodine intakes of mothers who had UIC < 150 μg/L were inadequate to raise iodine status to a level that provided optimal breast milk iodine for the infant.

The median maternal UIC of 107 μg/L at baseline indicated an improvement in the iodine status of the population compared with prior years because Ethiopia, including the study population, has been moderately to severely iodine deficient for decades (Median UIC in studies conducted in southern Ethiopia between 2007 and 2010 ranged between 1 μg/L to 89 μg/L) (Abuye & Kelbessa, 2000; Bogale et al., 2009; Ersino et al., 2013; Gebreegziabher et al., 2013; Girma et al., 2012). Therefore, the higher UIC observed at baseline in this study can be attributed to the salt iodization program that began to be implemented in 2012 in the country (Ethiopian Public Health Institute 2014).

The proportion of women with UIC below 100 μg/L decreased from 42% to 32% in the capsule group and from 53% to 45% in the I‐salt group between baseline and the end of the study. A double‐blind, randomized, placebo‐controlled study in Morocco reported a 14% increase in UIC at 6 months after giving the lactating mother a 400 mg iodized oil capsule (Bouhouch et al., 2014). The study in New Zealand showed that a daily supplement of either 75 μg or 150 μg of iodine daily for 6 months during lactation increased median maternal UIC significantly but the median values still remained below 100 μg/L (Mulrine et al., 2010).

In the current study, although the UIC increase was not significant, the proportion of women with the UIC above 100 μg/L was increased from 58% at baseline to 68% at 6 months in the capsule group and from 47% at baseline to 55% at 6 months in the I‐salt group.

The recommended dietary intakes (225–350 μg/day) of iodine for lactating mothers and 90 μg of iodine daily for neonates and infants are expected to produce UIC output in the neonates and infants of 180–225 μg/L (Delange, 2007). In the current study median UIC of infants was within the range of this expected output in both groups and at both times. Surprisingly the median UIC of infants obtained in this study at baseline was relatively high (218 μg/L) compared with data reported from three of four iodine sufficient countries (Zimmermann, 2007). However, despite the median UIC of 218 μg/L in our study, 17% of the infants at baseline and 16% at 6 months had UIC below 100 μg/L.

The median infant UIC at baseline was significantly higher than BMIC (*p* < .001) and maternal UIC (*p* < .001) and, although not significant, median BMIC tended to be higher than maternal UIC. This pattern was similar to other studies that reported these variables (Bouhouch et al., 2014; Mulrine et al., 2010). The majority of iodine consumed by lactating women is excreted in the breast milk; infants need small amounts to meet their daily requirement, and the remaining iodine will be excreted in their urine supporting this pattern (Delange, 2007; Zimmermann, 2007).

The correlation between maternal UIC and BMIC increased when maternal UIC was adjusted for creatinine. A study conducted in Denmark likewise reported that urinary iodine predicted the BMIC more precisely when it was adjusted for creatinine (Andersen, Moller, & Laurberg, 2014). Another study reported that urinary iodine was not associated with median thyroid stimulating hormone (TSH) and thyroxine (T_4_) but was significantly associated when it was adjusted for creatinine (Haddow, McClain, Palomaki, & Hollowell, 2007).

In our regression model for infant UIC at baseline, maternal urinary iodine adjusted for creatinine and BMIC were positive predictors and being female was a negative predictor. At 6 months maternal urinary iodine adjusted for creatinine and BMIC were strong positive predictors.

We did not analyze iodine content of complementary foods. When complementary feeding is begun, breast milk consumption will be reduced and the amount of iodine provided to the infant through breast milk will decrease (WHO 1991). In this study iodine concentration of all of the water samples from different sources was below the detection limit of 1 μg/L (Data not shown) predicting low iodine intake in the study area. Because these subsistence farmers do not buy foods from outside, iodized salt is a critical source of iodine for the study population. As infants begin to get a higher percent of energy from low iodine complementary foods, their total iodine intake will go down. In the study region (Southern Nations, Nationalities and Peoples’ Region), the median duration of exclusive breastfeeding was 2.2 months (Central Statistical Agency and ICF International 2012), so iodine intake of infants may begin to decrease early in life.

Unique strengths of the present study were that we recruited all of the women who delivered within the data collection time period and compliance was monitored by daily administration of capsules and weekly delivery of household salt. Unfortunately, obtaining approvals for the study caused baseline data collection to begin after iodized salt had begun to reach the market.

## CONCLUSIONS

In conclusion, our findings suggest that salt iodized at the recommended level (30 – 40 μg I as KIO_3_/g) provided to the household had a similar effect on BMIC and maternal and infant UIC as a daily supplement of 225 μg I as potassium iodide to lactating women. The iodine concentration of BMIC and UIC obtained in the study participants was comparable to median values in iodine sufficient countries (Zimmermann, 2007).

As a way to combat iodine deficiency disorders, universal salt iodization has been recommended. However, it is important to ensure that the salt is homogenously iodized and contains the required amount of iodine. The salt used in this study was iodized specifically for research purposes. The salt sold in the Ethiopian market at the time of the study varied notably in a range of 0–42 ppm (data not shown) which would result in variable iodine intakes.

## AUTHOR CONTRIBUTIONS

TG was involved in designing the study, data collection, laboratory and data analyses, and writing the article. BJS was involved in designing the study, laboratory and data analyses and writing the manuscript.

## CONFLICT OF INTEREST

The authors declare that they have no conflict of interest.

## References

[fsn3477-bib-0001] Abuye, C. , Berhane, Y. , Akalu, G. , Getahun, Z. , & Ersumo, T. (2007). Prevalence of goiter in children 6 to 12 years of age in Ethiopia. Food and Nutrition Bulletin, 28, 391–397. 18274165 10.1177/156482650702800403

[fsn3477-bib-0002] Abuye, C. , & Kelbessa, U. (2000). Determinants of iodine deficiency in school children in different regions of Ethiopia. East African Medical Journal, 77, 133–137. 12858887 10.4314/eamj.v77i3.46608

[fsn3477-bib-0003] Andersen, S. L. , Moller, M. , & Laurberg, P. (2014). Iodine concentrations in milk and in urine during breastfeeding are differently affected by maternal fluid intake. Thyroid, 24, 764–772. 24199933 10.1089/thy.2013.0541

[fsn3477-bib-0004] Andersson, M. , de Benoist, B. , Delange, F. , & Zupan, J. (2007). Prevention and control of iodine deficiency in pregnant and lactating women and in children less than 2‐years‐old: Conclusions and recommendations of the technical consultation. Public Health Nutrition, 10, 1606–1611. 18053287 10.1017/S1368980007361004

[fsn3477-bib-0005] Azizi, F. , & Smyth, P. (2009). Breastfeeding and maternal and infant iodine nutrition. Clinical Endocrinology, 70, 803–809. 19178515 10.1111/j.1365-2265.2008.03442.x

[fsn3477-bib-0006] Bogale, A. , Abebe, Y. , Stoecker, B. J. , Abuye, C. , Ketema, K. , & Hambidge, K. M. (2009). Iodine status and cognitive function of women and their five year‐old children in rural Sidama, southern Ethiopia. East African Journal of Public Health, 6, 296–299. 20803922

[fsn3477-bib-0007] Bouhouch, R. R. , Bouhouch, S. , Cherkaoui, M. , Aboussad, A. , Stinca, S. , Haldimann, M. , Andersson, M. , & Zimmermann, M. B. (2014). Direct iodine supplementation of infants versus supplementation of their breastfeeding mothers: A double‐blind, randomised, placebo‐controlled trial. The Lancet. Diabetes & Endocrinology, 2, 197–209. 24622750 10.1016/S2213-8587(13)70155-4

[fsn3477-bib-0008] Caldwell, K. L. , Maxwell, C. B. , Makhmudov, A. , Pino, S. , Braverman, L. E. , Jones, R. L. , & Hollowell, J. G. (2003). Use of inductively coupled plasma mass spectrometry to measure urinary iodine in NHANES 2000: Comparison with previous method. Clinical Chemistry, 49, 1019–1021. 12766019 10.1373/49.6.1019

[fsn3477-bib-0009] Central Statistical Agency and ICF International (2012). Ethiopia Demographic and Health Survey 2011. Addis Ababa, Ethiopia and Calverton, Maryland, USA: Central Statistical Agency and ICF International.

[fsn3477-bib-0010] Chan, S. , & Kilby, M. D. (2000). Thyroid hormone and central nervous system development. Journal of Endocrinology, 165, 1–8. 10750030 10.1677/joe.0.1650001

[fsn3477-bib-0011] Delange, F. (1998). Screening for congenital hypothyroidism used as an indicator of the degree of iodine deficiency and of its control. Thyroid, 8, 1185–1192. 9920376 10.1089/thy.1998.8.1185

[fsn3477-bib-0012] Delange, F. (2004). Optimal iodine nutrition during pregnancy, lactation and the neonatal period. International Journal of Endocrinology and Metabolism, 2, 1–12.

[fsn3477-bib-0013] Delange, F. (2007). Iodine requirements during pregnancy, lactation and the neonatal period and indicators of optimal iodine nutrition. Public Health Nutrition, 10, 1571–1580; discussion 1581‐1573. 18053281 10.1017/S1368980007360941

[fsn3477-bib-0014] Dorea, J. G. (2002). Iodine nutrition and breast feeding. Journal of Trace Elements in Medicine and Biology, 16, 207–220. 12530582 10.1016/S0946-672X(02)80047-5

[fsn3477-bib-0015] Ersino, G. , Tadele, H. , Bogale, A. , Abuye, C. , & Stoecker, B. J. (2013). Clinical assessment of goiter and low urinary iodine concentration depict presence of severe iodine deficiency in pregnant Ethiopian women: A cross‐sectional study in rural Sidama, southern Ethiopia. Ethiopian Medical Journal, 51, 133–141. 24079157

[fsn3477-bib-0016] Ethiopian Public Health Institute (2014) National salt iodization coverage towards prevention of iodine deficiency disorders in Ethiopia. (A report)

[fsn3477-bib-0017] Fecher, P. A. , Goldmann, I. , & Nagengast, A. (1998). Determination of iodine in food samples by inductively coupled plasma mass spectrometry after alkaline extraction. Journal of Analytical Atomic Spectrometry, 13, 977–982.

[fsn3477-bib-0018] Gebreegziabher, T. , Teyike, N. , Mulugeta, A. , Abebe, Y. , Hambidge, K. M. , & Stoecker, B. J. (2013). Lack of dietary sources of iodine and the prevalence of iodine deficiency in rural women from Sidama zone, southern Ethiopia. African Journal of Food, Agriculture, Nutrition and Development, 13, 8401–8414.

[fsn3477-bib-0019] Girma, M. , Loha, E. , Bogale, A. , Teyike, N. , Abuye, C. , & Stoecker, B. J. (2012). Iodine deficiency in primary school children and knowledge of iodine deficiency and iodized salt among caretakers in Hawassa town: Southern Ethiopia. Ethiopian Journal of Health Development, 26, 30–35.

[fsn3477-bib-0020] Haddow, J. E. , McClain, M. R. , Palomaki, G. E. , & Hollowell, J. G. (2007). Urine iodine measurements, creatinine adjustment, and thyroid deficiency in an adult United States population. Journal of Clinical Endocrinology and Metabolism, 92, 1019–1022. 17200163 10.1210/jc.2006-2156

[fsn3477-bib-0021] Halpern, J. , Boyages, S. C. , Maberly, G. F. , Collins, J. K. , Eastman, C. J. , & Morris, J. G. L. (1991). The neurology of endemic cretinism: A study of two endemias. Brain, 114, 825–841. 2043952 10.1093/brain/114.2.825

[fsn3477-bib-0022] Institute of Medicine (2001). Dietary reference intakes for Vitamin A, Vitamin K, Arsenic, Boron, Chromium, Copper, Iodine, Iron, Manganese, Molybdenum, Nickel, Silicon, Vanadium and Zinc. Washington DC: National Academy Press. 25057538

[fsn3477-bib-0023] Leung, A. M. , Braverman, L. E. , He, X. , Heeren, T. , & Pearce, E. N. (2012). Breastmilk iodine concentrations following acute dietary iodine intake. Thyroid, 22, 1176–1180. 23050787 10.1089/thy.2012.0294 PMC3487113

[fsn3477-bib-0024] Mulrine, H. M. , Skeaff, S. A. , Ferguson, E. L. , Gray, A. R. , & Valeix, P. (2010). Breast milk iodine concentration declines over the first 6 mo postpartum in iodine‐deficient women. American Journal of Clinical Nutrition, 92, 849–856. 20702609 10.3945/ajcn.2010.29630

[fsn3477-bib-0025] Neville, M. C. , Keller, R. , Seacat, J. , Lutes, V. , Neifert, M. , Casey, C. , Allen, J. , & Archer, P. (1988). Studies in human lactation: Milk volumes in lactating women during the onset of lactation and full lactation. American Journal of Clinical Nutrition, 48, 1375–1386. 3202087 10.1093/ajcn/48.6.1375

[fsn3477-bib-0026] Pharoah, P. O. D. , Buttfield, I. H. , & Hetzel, B. S. (1971). Neurological damage to the foetus resulting from severe iodine deficiency during pregnancy. Lancet, 1, 308–310. 4100150 10.1016/s0140-6736(71)91040-3

[fsn3477-bib-0027] Rhee, S. S. , Braverman, L. E. , Pino, S. , He, X. , & Pearce, E. N. (2011). High iodine content of Korean seaweed soup: A health risk for lactating women and their infants? Thyroid, 21, 927–928. 21745110 10.1089/thy.2011.0084

[fsn3477-bib-0028] Semba, R. D. , & Delange, F. (2001). Iodine in human milk: Perspectives for infant health. Nutrition Reviews, 59, 269–278. 11518182 10.1111/j.1753-4887.2001.tb05512.x

[fsn3477-bib-0029] Spitzweg, C. , Joba, W. , Eisenmenger, W. , & Heufelder, A. E. (1998). Analysis of human sodium iodide symporter gene expression in extrathyroidal tissues and cloning of its complementary deoxyribonucleic acids from salivary gland, mammary gland, and gastric mucosa. Journal of Clinical Endocrinology and Metabolism, 83, 1746–1751. 9589686 10.1210/jcem.83.5.4839

[fsn3477-bib-0030] Tazebay, U. H. , Wapnir, I. L. , Levy, O. , Dohan, O. , Zuckier, L. S. , Zhao, Q. H. , Fu Deng, H. , Amenta, P. S. , Fineberg, S. , Pestell, R. G. , & Carrasco, N. (2000). The mammary gland iodide transporter is expressed during lactation and in breast cancer. Nature Medicine, 6, 871–878. 10.1038/78630 10932223

[fsn3477-bib-0031] WHO (1991). Indicators for assessing breastfeeding practices. Division of Child Health and Development. (WHO/CDD/SER 91.14).

[fsn3477-bib-0032] WHO (1998). Complementary feeding of young children in developing countries: A review of current scientific knowledge. Geneva, World Health Organization. (WHO/NUT/98.1).

[fsn3477-bib-0033] WHO, UNICEF (2007). Reaching optimal iodine nutrition in pregnant and lactating women and young children: Programmatic recommendations. Public Health Nutrition, 10(12A), 1527–1529. 18333291 10.1017/s1368980007705360

[fsn3477-bib-0034] WHO/UNICEF/ICCIDD (1996). Recommended iodine levels in salt and guidelines for monitoring their adequacy and effectiveness. Geneva, World Health Organization. (WHO/NUT/96.13).

[fsn3477-bib-0035] WHO/UNICEF/ICCIDD (2001). Assessment of iodine deficiency disorders and monitoring their elimination. 2nd ed. Geneva, World Health Organization. (WHO/NHD/01.1, 1‐107).

[fsn3477-bib-0036] WHO/UNICEF/ICCIDD (2007). Assessment of iodine deficiency disorders and monitoring their elimination. a guide for program managers. 3rd ed. Geneva, World Health Organization.

[fsn3477-bib-0037] Zimmermann, M. B. (2007). The impact of iodised salt or iodine supplements on iodine status during pregnancy, lactation and infancy. Public Health Nutrition, 10, 1584–1595. 18053283 10.1017/S1368980007360965

[fsn3477-bib-0038] Zimmermann, M. B. (2009). Iodine deficiency. Endocrine Reviews, 30, 376–408. 19460960 10.1210/er.2009-0011

